# Carbon‐Ion Radiation Therapy Compared to Surgical Resection for Primary, Solitary, Potentially Resectable Hepatocellular Carcinoma

**DOI:** 10.1002/jgh3.70256

**Published:** 2025-09-23

**Authors:** Soichiro Morinaga, Shinnosuke Kawahara, Rei Kanemoto, Naohiko Matsushita, Yuto Kamioka, Mariko Kamiya, Masaaki Murakawa, Taito Fukushima, Satoshi Kobayashi, Makoto Ueno, Hiroyuki Kato, Naoto Yamamoto

**Affiliations:** ^1^ Department of Hepato‐Biliary and Pancreatic Surgery Kanagawa Cancer Center Yokohama Japan; ^2^ Department of Gastroenterology Kanagawa Cancer Center Yokohama Japan; ^3^ Department of Radiation Oncology Kanagawa Cancer Center Yokohama Japan

**Keywords:** carbon‐ion radiation therapy, hepatocellular carcinoma, liver surgery, radiation therapy

## Abstract

**Aims:**

Carbon‐ion radiation therapy (CIRT) is a promising technological innovation for treating hepatocellular carcinoma (HCC). This study aimed to evaluate the effectiveness and safety of CIRT for primary, solitary, potentially resectable HCC in comparison to liver resection (LR).

**Methods and Results:**

We retrospectively compared treatment effectiveness and safety between patients treated with CIRT and those who underwent LR for potentially resectable HCC at our institution. The clinical data for the CIRT group were obtained from a prospective observational study carried out at our institution, and additional information was obtained from clinical records. Their data were compared with those of patients who underwent LR during the same period. Twenty‐three patients were included in the CIRT group and 41 in the LR group. In the overall cohort, the respective 3‐and 5‐year overall survival (OS) rates were 86.5% and 65.9% for the CIRT group and 90.2% and 79.7% for the LR group. The OS rates did not significantly differ between the two groups in the propensity score‐matched cohort. The 3‐ and 5‐year local control rates after CIRT were 77.0% and 77.0%, respectively. CIRT was associated with elevated albumin‐bilirubin (ALBI) scores 3 and 6 months after treatment.

**Conclusion:**

CIRT for primary, solitary, potentially resectable HCC was associated with favorable clinical outcomes and satisfactory safety, with an acceptable elevation of the ALBI score. CIRT might achieve a favorable OS comparable to LR for potentially resectable HCC; however, further large‐scale, prospective studies are needed for confirmation.

## 1 Introduction

Liver cancer is the sixth most common cancer and the third most common cause of cancer‐related death worldwide [1]. Hepatocellular carcinoma (HCC) accounts for 75% of all cases of primary liver cancer [2]. It is an aggressive tumor that often occurs in patients with underlying cirrhosis or chronic liver diseases, mainly related to infections with hepatitis B virus (HBV) and/or hepatitis C virus, chronic alcohol consumption, or nonalcoholic fatty liver disease [2].

The treatment of patients with HCC is determined by considering both the extent of the tumor and the severity of liver dysfunction caused by underlying liver diseases. Treatment options for patients with localized disease include surgical resection, transplantation, ablation, arterially directed therapies, and external‐beam radiation therapy (EBRT) [3, 4]. When patients are eligible for resection based on the tumor extent and their liver functional reserve, potentially curative resection is preferable to other therapies; the relative effectiveness of other therapies compared to resection has not been established [3, 4, 5, 6, 7]. Notably, ablation is a viable first‐line option for patients with small (< 3 cm) and appropriately located tumors [3, 4].

EBRT has been underutilized as an HCC treatment because of its toxicity to the surrounding liver tissue [8]. A sufficient radiation dose must be delivered to kill the tumors while the dose to the surrounding liver must be minimized, which is difficult to achieve using traditional technologies. Charged‐particle therapy is a promising modern technological innovation that addresses this concern. Using proton or carbon ions instead of photons enables the accurate delivery of high‐intensity radiation to deep‐seated tumors while sparing the surrounding, radiosensitive tissue [8, 9]. The physical characteristics of such particles provide for a favorable radiation dose distribution, marked by a low entrance dose, sharp accumulation with a steep dose fall‐off at the end of the beam range (Bragg peak), and a small exit dose beyond the target [9, 10, 11, 12]. In addition, as carbon ions are heavier than protons, carbon‐ion radiation therapy (CIRT) increases the rate of cell death owing to double‐strand DNA breaks, which are challenging to repair via cellular repair mechanisms, consequently producing a higher linear energy transfer and superior relative biological effectiveness (RBE) [9, 10, 11, 12]. On the other hand, due to the high initial construction costs and operational expenses, the number of heavy ion therapy facilities is limited, making it difficult for patients to access them easily.

Evidence for the efficacy and safety of CIRT for HCC has been accumulating [13, 14]. Previous retrospective reports including patients with various extents and stages of tumors showed the 3‐ and 5‐year local‐control rates of 76.5%–95.7% and 81.0%–95.7%, and the 3‐and 5‐year survival rates of 44.4%–76.7% and 22.2%–36.8%, respectively, with no severe adverse events (AEs) [15, 16, 17, 18, 19, 20]. Recent retrospective comparative studies have shown that CIRT provides better local control, overall survival (OS), and progression‐free survival in treatment‐naïve patients with a single HCC compared to trans‐arterial therapy [21], and comparable local control and OS to radiofrequency ablation (RFA) when used as an initial treatment for early‐stage HCC [22]. However, comparing treatment efficacy between CIRT and surgical resection remains unaddressed.

Given that CIRT is an evolving technology that yields excellent local control and long‐term outcomes as treatment for HCC, the aim of this study was to evaluate the clinical outcomes of patients treated with CIRT for primary, solitary, potentially resectable HCC compared with those of patients treated with surgical resection.

## 2 Patients and Methods

### 2.1 CIRT Group

**FIGURE 1:**
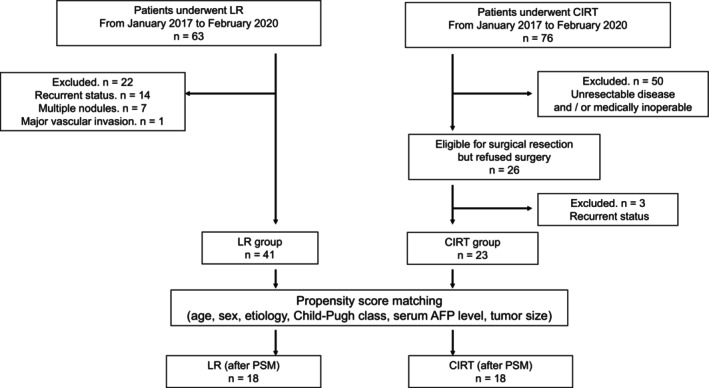
Flow diagram of patient selection and matching criteria. AFP, alpha‐fetoprotein; CIRT, carbon‐ion radiation therapy; LR, liver resection; PSM, propensity score matching.

From January 2017 to February 2020, 76 patients underwent CIRT for HCC at our institution. Fifty of them had unresectable disease owing to tumor extent or poor liver functional reserve and/or were considered medically inoperable owing to comorbidities. The remaining 26 patients were registered in the iROCK‐1601LI study, a prospective observational study conducted at our institution for patients who declined surgical resection despite being deemed eligible for surgical resection by a multidisciplinary team and opted for CIRT as HCC treatment. The inclusion criteria for this study were as follows: a solitary lesion of any size without major vascular invasion, primary or recurrent status, Child‐Pugh class A or B (score ≤ 9) liver function, an Eastern Cooperative Oncology Group Performance Status of 0–2, and sufficiently preserved cardiac, respiratory, and renal functions. The primary endpoint was the 3‐year local control rate, and the secondary endpoints were OS and AEs. Additional data were obtained from their clinical records for the present study. Three of these patients were excluded from the present study owing to recurrent status. Finally, 23 patients were included in the CIRT group (Figure 1).

### 2.2 Liver‐Resection Group

From January 2017 to February 2020, 63 patients underwent liver resection (LR) for HCC at our institution. Fourteen of these were excluded owing to recurrent disease, seven owing to multiple nodules, and one owing to major vascular invasion. Finally, 41 patients who underwent resection for primary, solitary tumors of any size without major vascular invasion were included in the LR group (Figure 1). All patients had well‐preserved liver function (Child–Pugh class A or B), satisfied Makuuchi's selection criteria [23], and were medically indicated for surgery. Indications for surgical resection were determined via a multidisciplinary review. Anatomical or non‐anatomical resection was performed based on the tumor extent, tumor location, and liver function, with the intent to achieve negative surgical margins. Both open and laparoscopic LR were performed. During the study period, laparoscopic LR was indicated for partial hepatectomy or lateral segmentectomies.

### 2.3 Diagnostic Imaging

Patients included in this study were evaluated with dynamic contrast‐enhanced computed tomography (CT) and/or magnetic resonance imaging (MRI) in the 28 days before diagnosis. An HCC lesion was defined as a lesion characterized by arterial hypervascularity and washout in the portal venous phases.

### 2.4 CIRT

Metallic fiducial markers were implanted beside the target tumor as landmarks. Contrast‐enhanced CT (2 mm sections) was performed for optimal target definition during treatment planning. The gross tumor volume (GTV) was defined as the area of solid, macroscopic contrast enhancement of the tumor upon CT. The clinical target volume (CTV) was defined as the GTV plus a margin of 5–10 mm, with modifications to exclude the gastrointestinal tract and portal vein. The planning target volume (PTV) included the CTV plus a margin of 5 mm, accounting for organ motion and setup inaccuracies. The PTV was modified to exclude at‐risk organs. The treatment plan aimed to cover 100% of the PTV with 95% of the prescribed dose. Treatment plans for CIRT were created using Monaco software, version 5.20, for carbon‐ion scanning (Elekta AB, Stockholm, Sweden). Respiratory‐gated irradiation was performed after the patient was appropriately positioned. A total dose of 60.0 Gy (RBE) was delivered in 4 or 12 fractions, depending on the tumor size and location. The CIRT was performed on an outpatient basis.

### 2.5 Follow‐Up

Patients were followed up every 3 months after treatment with blood tests and serum alpha‐fetoprotein (AFP) testing. In addition, contrast‐enhanced CT or magnetic resonance imaging was performed every 3 months for the first 6 months and every 3–6 months thereafter. CIRT‐related AEs were evaluated using the Common Terminology Criteria for Adverse Events (CTCAE), version 4.0. Postoperative complications were defined using Clavien–Dindo (C‐D) classification [24]. Recurrence was defined based on radiological and histological observations and categorized as local, liver, or distant recurrence. Local recurrence was defined as the regrowth or appearance of a new lesion in the radiation field or resection margin. Liver recurrence was defined as recurrence in the remnant liver, either via intrahepatic metastasis or as a newly developed tumor. Distant recurrence was defined as extrahepatic recurrence. Patients with recurrent disease were treated with either locoregional or systemic therapy according to the disease extent, remnant liver function, and medical condition.

### 2.6 Evaluation of Liver Function After Treatment

To evaluate serial changes in remnant liver function, the ALBI score and PT‐INR were calculated before treatment and at 3 and 6 months following CIRT or LR.

### 2.7 PSM Analysis

Propensity score‐matching (PSM) analysis was performed to eliminate baseline differences between the two groups. PSM was performed for age, sex, etiology, Child–Pugh class, serum AFP level, and tumor size. Propensity scores were generated using multivariable logistic regression, and one‐to‐one nearest‐neighbor matching without replacement was performed with a caliper width of < 0.2 of the pooled standard deviation of estimated propensity scores. IBM SPSS Statistics for Windows (version 25; IBM Corporation, Armonk, NY, USA) was used for all analyses.

### 2.8 Statistical Analyses

Categorical data are presented as counts and percentages and were compared using Fisher's exact test. Continuous variables are described as medians and ranges and were compared using the Mann–Whitney *U* test. Paired samples were compared using the Wilcoxon test. The cumulative disease‐free survival (DFS), OS, and local‐control rates were estimated using the Kaplan–Meier method and compared using the log‐rank and generalized Wilcoxon tests. To evaluate factors associated with OS and DFS, multivariate analysis was carried out using the Cox proportional hazards regression model. DFS and OS were calculated from the date of surgery or the date of CIRT initiation. The significance level was set at *p* < 0.05 for all statistical analyses. IBM SPSS Statistics for Windows (version 25; IBM Corporation) was used for statistical analyses.

## 3 Ethics

Informed consent was obtained from all patients to use their clinical data for this study, according to the institutional guidelines of the Kanagawa Cancer Center. This study conformed to the ethical guidelines of the Declaration of Helsinki and was approved by the Institutional Review Board of Kanagawa Cancer Center (approval no.: 2023 study 19). The iROCK‐1601LI study conformed to the ethical guidelines of the Declaration of Helsinki and was approved by the Institutional Review Board of Kanagawa Cancer Center (approval no.: 27‐study‐63).

## 4 Results

### 4.1 Patient Characteristics

Table 1 summarizes the baseline characteristics of the patients before and after PSM. Before matching, patients in the CIRT group were significantly older than those in the LR group, but the groups did not significantly differ in terms of sex, tumor size, Child–Pugh class, albumin‐bilirubin (ALBI) grade, or etiology. After matching, no statistically significant differences were observed between the two groups, with 18 patients in each group.

**TABLE 1:** Baseline characteristics before and after propensity score matching.

	Before matching			After matching		
	CIRT *n* = 23	LR *n* = 41	*p*	CIRT (*n* = 18)	LR (*n* = 18)	*p*
Age, years median (range)	77.0 (51–94)	71.0 (41–83)	0.028	74.5 (51–87)	71.0 (53–83)	0.481
Gender						
Male/female number	20/3	37/4	0.689	18/0	17/1	> 0.99
Etiology number (%)						
HCV	6 (26.1)	7 (17.1)	0.613	4 (22.2)	3 (16.7)	0.896
HBV	7 (30.4)	10 (24.4)		6 (33.3)	7 (38.9)	
HCV + HBV	0	1 (2.4)		0	0	
Others	10 (43.5)	23 (56.1)		8 (44.4)	08 (44.4)	
Child‐Pugh						
A/B number	23/0	40/1	1.000	18/0	18/0	> 0.99
AFP ng/mL median (range)	4.1 (1.1–801.0)	6.4 (1.0–108.000)	0.122	3.9 (1.1–801.0)	4.1 (1.0–63.4)	0.815
Tumor size mm median (range)	35.0 (10.0–110.0)	35.0 (12.0–123.0)	0.561	35.0 (20.2–110.0)	38.5 (12.0–123.0)	0.606
ALBI grade						
Grade 1	20	33	0.73^a^	16	13	0.402^a^
Grade 2 (2a/2b)	3 (3/0)	8 (4/4)		2 (2/0)	5 (4/1)	
Complications						
≥ Grade 3	1 (4%)	3 (7%)	0.638	NA	NA	NA
	CTCAE	CD				

Abbreviations: AFP, alpha‐fetoprotein; ALBI, Albumin‐Bilirubin; CD, Clavien‐Dindo; CIRT, carbon ion radiation therapy; CTCAE, Common Terminology Criteria for Adverse Events; HBV, hepatitis B virus; HCV, hepatitis C virus; LR, liver resection; NA, not assessed.

^a^ Fisher's exact test (grade 1 vs. grade 2).

### 4.2 OS and DFS Before and After PSM

**FIGURE 2:**
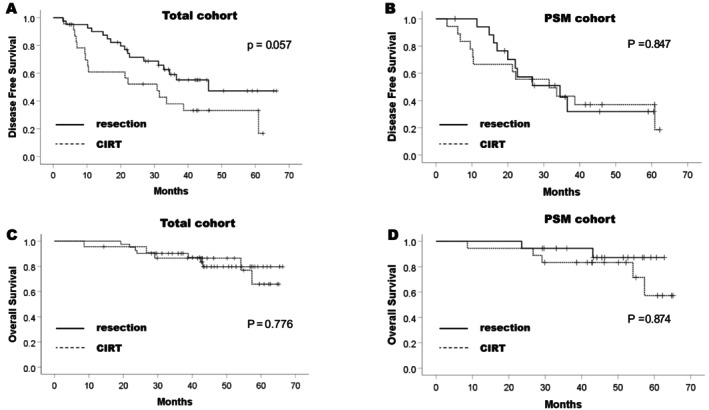
Cumulative disease‐free and overall survival curves for the liver resection and CIRT groups, in the total cohort (A, C) and the PSM cohort (B, D). *p*‐values are estimated by log‐rank test. CIRT, carbon‐ion radiation therapy; PSM, propensity score matching.

Figure 2 displays the cumulative OS and DFS curves before and after PSM. In the overall cohort, after a median follow‐up period of 43.9 (range, 8.6–66.3) months, the estimated 3‐ and 5‐year OS rates were 86.5% and 65.9%, respectively, for the CIRT group and 90.2% and 79.7%, respectively, for the LR group. The OS curves did not significantly differ between the two groups by log‐rank test (*p* = 0.776). After a median follow‐up time of 31.2 (2.8–66.2) months, the estimated 3‐ and 5‐year DFS rates were 37.9% and 33.2%, respectively, for the CIRT group and 59.1% and 47.3%, respectively, for the LR group. The DFS curves did not reach statistical difference by log‐rank test (*p* = 0.057).

After PSM, the estimated 3‐ and 5‐year OS rates were 83.3% and 57.1%, respectively, for the CIRT group and 94.4% and 87.2%, respectively, for the LR group. The OS curves showed no statistical difference (log‐rank test, *p* = 0.874). The estimated 3‐ and 5‐year DFS rates after PSM were 43.2% and 37.0%, respectively, in the CIRT group and 42.5% and 31.9%, respectively, in the LR group. The DFS curves showed no statistical difference (log‐rank test, *p* = 0.847).

We used a generalized Wilcoxon test to estimate the initial treatment effects on survival in shorter survival times on the Kaplan–Meier curves. In the overall cohort, the OS curves did not significantly differ between the two groups (*p* = 0.972); but CIRT yielded significantly poorer DFS compared with LR (*p* = 0.041).

Upon multivariate analysis (Table 2), which includes the treatment procedure, age, AFP level, tumor size, and ALBI grade, tumor size was the factor most likely associated with OS; however, it did not reach statistical significance (HR 6.006; *p* = 0.088).

**TABLE 2:** Multivariate analysis of predictive factors for OS.

Factor	Hazard ratio	95% CI	*p*
CIRT vs. LR	1.054	0.328–3.394	0.929
ALBI Grade 1 vs. Grade 2	2.708	0.653–11.237	0.170
AFP (ng/mL) ≤ 20 vs. > 20	1.177	0.290–4.780	0.819
Age (years) ≤ 70 vs. > 70	2.637	0.598–11.638	0.201
Size (mm) ≤ 30 vs. > 30	6.006	0.765–47.140	0.088

Abbreviations: AFP, alpha‐fetoprotein; ALBI, albumin bilirubin; CI, confidence interval; CIRT, carbon ion radiation therapy; LR, liver resection.

### 4.3 Local‐Control Rate

**FIGURE 3:**
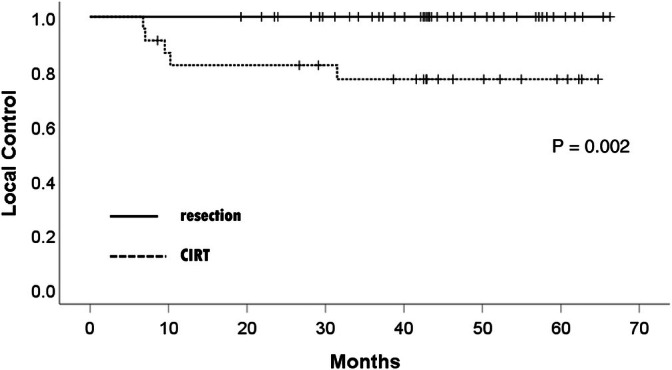
Cumulative local control rate after liver resection and CIRT. CIRT, carbon‐ion radiation therapy.

Figure 3 illustrates the cumulative local‐control rates after CIRT and LR in the overall cohort.

Four patients experienced only local recurrence, and one experienced both local and distant recurrence after CIRT. The local‐control rates after CIRT at 1, 3, and 5 years were 82.2%, 77.0%, and 77.0%, respectively; but no patients experienced local recurrence after LR.

### 4.4 Initial Recurrence Site

Table 3 summarizes the sites of initial recurrence. After CIRT, four patients (17%) experienced only local recurrence; nine (39%) experienced recurrence in the remnant liver, two (9%) experienced recurrences in the lungs, and one experienced simultaneous local and distant recurrence (in a lung). After LR, 16 patients (39%) experienced recurrence in the remnant liver, and one (4%) experienced it in the peritoneum. The CIRT group had a higher local recurrence rate; however, the remnant liver recurrence and distant recurrence rates did not differ between the groups.

**TABLE 3:** Initial recurrence site.

Recurrence site	CIRT	LR	*p*
Local	4/23 (17%)	0/41	0.004
Liver	9/23 (39%)	16/41 (39%)	> 0.99
Distant			
Lung	2/23 (9%)	0/41	
Peritoneal	0/23	1/41 (2%)	0.128
Both local and distant (lung)	1/23 (4)	0/41	NA

*Note:* Data are displayed as *n* (%).

Abbreviations: CIRT, carbon‐ion radiation therapy; LR, liver resection; NA, not assessed.

### 4.5 Treatment for Initial Recurrence

Among the four patients who experienced only local recurrence after CIRT, one underwent subsequent LR, one underwent another round of CIRT, one underwent CIRT with induction trans‐arterial chemoembolization (TACE), and one underwent subsequent RFA. Among the nine patients who experienced recurrence in the remnant liver after CIRT, 4 patients underwent CIRT for the recurrence, one underwent RFA, one underwent TACE, one underwent systemic therapy, and 2 were treated with BSC. Among 16 patients who experienced recurrence in the remnant liver after LR, one underwent LR, 9 underwent RFA, 4 underwent TACE, one underwent systemic therapy for the recurrence, and one was treated with BSC.

### 4.6 AEs

None of the patients experienced CTCAE grade ≥ 4 toxicity after CIRT. One patient developed grade 3 elevated liver enzyme levels (4%), and two patients developed grade 2 elevated liver enzyme levels (9%). One patient developed grade 2 lower‐intestinal bleeding, one developed grade 1 pleural effusion, and one developed grade 1 nausea. The most common AE was grade 1 radiation dermatitis (5/23; 22%).

After LR, none of the patients experienced C‐D grade ≥ IV complications. Three patients developed C‐D grade III intra‐abdominal abscesses (7%), three developed C‐D grade II ascites (5%), and two developed C‐D grade II pleural effusion (5%). No difference was observed in the incidence of CTCAE grade ≥ 3 toxicity after CIRT or in C‐D grade ≥ III complications after LR (Table 1).

### 4.7 Serial Changes in Liver Function Before and After Treatment for HCC

**FIGURE 4:**
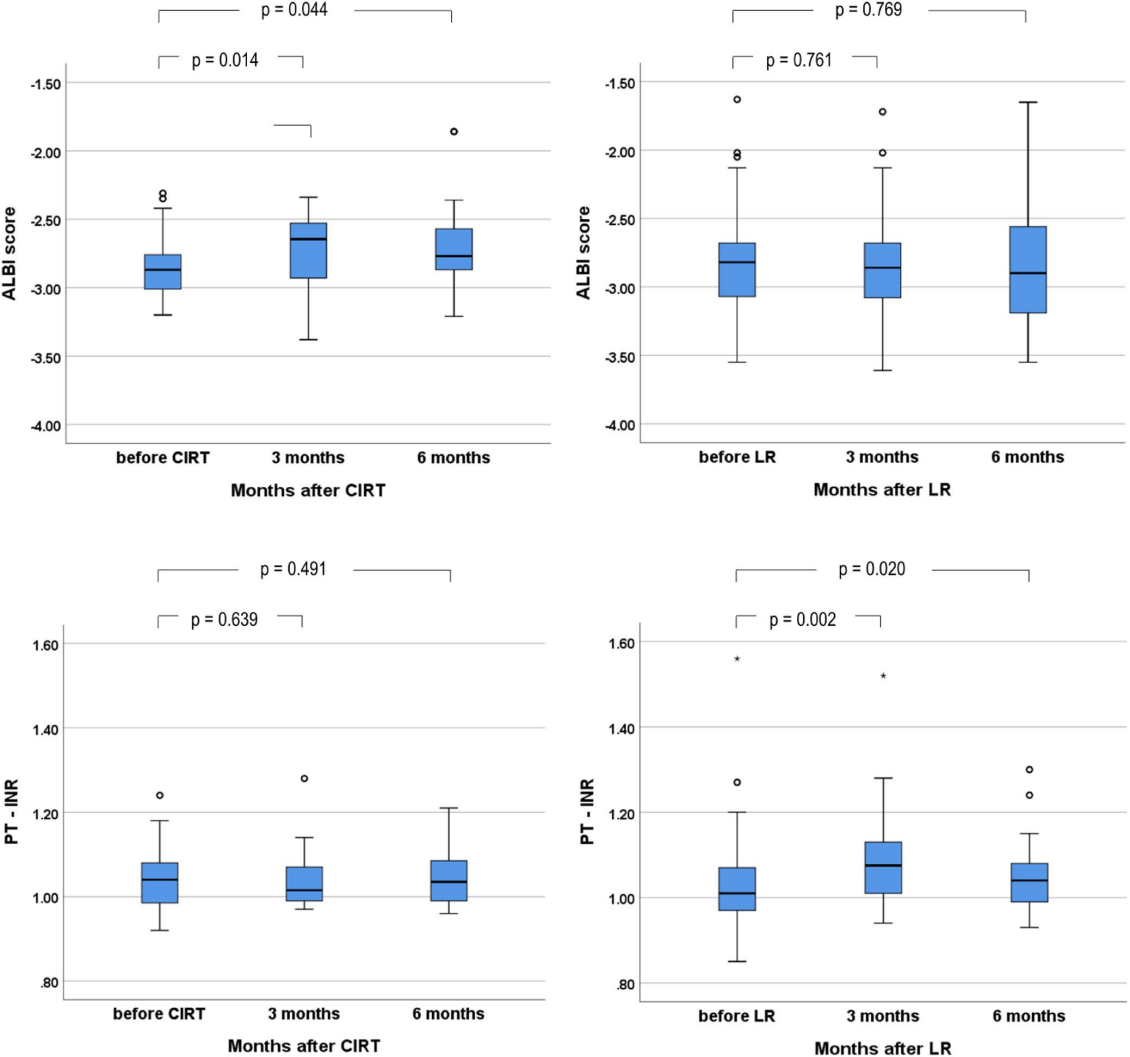
Serial changes in ALBI score and PT‐INR after treatment. ALBI score, Albumin‐Bilirubin score; CIRT, carbon‐ion radiation therapy; LR, liver resection; PT‐INR, prothrombin time‐international normalized ratio.

The CIRT group exhibited significant increases in the ALBI scores three (*p* = 0.014) and six (*p* = 0.044) months after treatment compared with that before treatment; but the LR group exhibited no differences. The LR group exhibited a significant increase in the PT‐INR three (*p* = 0.002) and six (*p* = 0.020) months after treatment; but the CIRT group exhibited no differences (Figure 4).

## 5 Discussion

In this study, we evaluated the treatment effectiveness and safety of CIRT for patients with primary, solitary, potentially resectable HCC and compared the results with those of LR. We discovered that CIRT was associated with favorable clinical outcomes and satisfactory safety, with an acceptable elevation in the ALBI score. Our study also suggested that CIRT for potentially resectable HCC might achieve an OS comparable to that of LR.

In the overall cohort of this study, patients treated via CIRT had 3‐ and 5‐year OS rates of 86.5% and 65.9%, respectively, which are more favorable than the previously reported 3‐year OS rates of 44.4%–76.7% and 5‐year OS rates of 22.2%–36.8% for CIRT in HCC patients with various tumor extents, tumor stages, and primary or recurrent tumors [15, 16, 17, 18, 19, 20]. On the other hand, two comparative studies reported similar OS rates following CIRT. One study comparing CIRT and TACE reported a 3‐year OS rate of 74% after CIRT in treatment‐naïve HCC patients with a single lesion, and no major vascular invasion or extrahepatic metastases [21]. Another study comparing CIRT and RFA reported a 2‐year OS rate of 83.7% and a 5‐year OS rate of 55.7% in patients with primary, early HCC (single lesion ≤ 5 cm or 2–3 lesions ≤ 3 cm), without macrovascular invasion or extrahepatic metastases, and with well‐preserved liver function [22].

Although this study has certain limitations, our results suggest that CIRT may provide an OS equivalent to LR in patients with primary, solitary, potentially resectable HCC. These OS rates of our patients were comparable to those reported for patients who underwent resection for HCC worldwide. LR yields 5‐year survival rates > 50%. Among carefully selected patients with early‐stage HCC and well‐preserved liver function, the 5‐year OS rate after LR is approximately 70% [3]. Patients with Barcelona Clinic Liver Cancer stage A disease in one study had 3‐ and 5‐year OS rates of 78% and 68%, respectively, after LR [25]. In another study, patients with a single HCC, ≤ 2 cm in size and preserved liver function (Child–Pugh class A) had a 5‐year OS rate of 70% after LR [26]. In addition, we detected no significant difference in the OS between patients treated with CIRT and those who underwent LR, either for all patients or after PSM.

Regarding local control, our data suggested the superiority of LR over CIRT. CIRT is an emerging radiotherapy modality for HCC, exhibiting a high linear energy transfer and RBE. However, tumors still recur in the radiation field. In our study, the 3‐year local‐recurrence rate was 23% among patients treated with CIRT, including four cases of local recurrence only and one of recurrence at both a local and distant site, which aligns with the 17.7% local‐recurrence rate after CIRT for patients with early‐stage HCC in one study [22]. In contrast, we experienced no local recurrence after LR in this study. Moreover, the two recent prospective randomized studies comparing surgical resection with RFA revealed local‐recurrence rates of 3.7% and 15% after surgical resection [27, 28]. These data suggest the superiority of LR over CIRT in terms of local control.

Local recurrence in the radiation field after CIRT may affect the DFS, particularly during the early post‐treatment period. To better estimate the initial treatment effect on short‐term outcomes, we performed the generalized Wilcoxon test for DFS in addition to the log‐rank test. The generalized Wilcoxon test places more weight on differences in shorter survival times of the survival curve, whereas the log‐rank test favors differences toward longer survival times [29]. In the present study, the generalized Wilcoxon test indicated that CIRT was associated with a lower DFS rate than LR, while the log‐rank test showed no significant difference in DFS between the two groups. HCC has a high risk of recurrence in the remnant liver, owing to either intrahepatic metastasis or newly developed HCC. In our study, approximately 40% of patients experienced recurrence in the remnant liver after CIRT or LR. Even after curative resection, HCC recurs in 50%–70% of patients within 5 years [4]. In a recent prospective, randomized trial of surgery versus RFA for early‐stage HCC [28], higher local recurrence rates were noted in the RFA group. However, this difference did not affect the long‐term DFS, which was comparable between the two groups. Whether local recurrence after CIRT affects the long‐term DFS remains, an issue that needs to be clarified in future studies.

As CIRT can deliver the radiation dose with higher intensity and accuracy, the surrounding liver tissue and liver function can be preserved [20]. We evaluated liver damage after treatment by measuring the serial changes in the ALBI score and the PT‐INR before, and 3 and 6 months after treatment. Both CIRT and LR were associated with functional damage to the liver, and to an acceptable degree. CIRT was associated with increased ALBI scores, and LR was associated with increased PT‐INR. The mechanism by which CIRT and LR affect liver function remains to be clarified in future studies.

The well‐preserved liver function enables provision of curative second‐line treatment for patients with local recurrence. As the rates of recurrence in the remnant liver or distant sites after CIRT were equal to those after LR, the availability of potentially curative second‐line treatment for local recurrence after CIRT may explain why the OS after CIRT was comparable to that after LR in patients with potentially resectable HCC in this study. In our study, four patients experienced only local recurrence after CIRT. Among them, one underwent another round of CIRT, one underwent CIRT with induction TACE, one underwent subsequent LR, and one underwent subsequent RFA. Of these four patients, one patient had another local recurrence after CIRT, one had remnant liver recurrence after RFA, and two had a distant recurrence for the lung after CIRT with induction TACE or surgical resection. These four patients achieved a median OS of 55.7 months (range 29.8–65.3 months) after initial treatment, comparable to 43.2 months (range 19.2–66.2 months) after LR for initial treatment.

This study has several limitations. While part of the data for the CIRT group was obtained from a prospective observational study conducted at our institution, the present study was retrospective and carried out in a single center. As CIRT for patients with HCC who were eligible for surgical resection was provided only for patients who declined surgery, the sample in this study was small. PSM was performed to minimize selection bias, which further reduced the number of cases. Our results should be validated in a prospective, multicenter controlled trial involving a larger number of patients.

Caution is needed when interpreting the results of this study. Currently, the use of CIRT for resectable HCC is limited to patients who are medically inoperable or those who refused surgical resection. The clinical guidelines recommend LR as the first‐line treatment for resectable HCC when deemed appropriate for resection based on the tumor extent and liver functional reserve [3, 4]. Since EBRT is recommended for patients with liver‐confined HCC who are not candidates for curative surgery or ablation therapy [30], CIRT would be an optimal alternative to LR for patients who are unable to undergo surgery due to comorbidities or other factors. It is safe, effective, and can be provided as outpatient care. The potential of CIRT as a first‐line treatment for resectable HCC warrants further evaluation in future studies. Currently, the Japan Clinical Oncology Group (https://jcog.jp) is conducting a prospective, multicenter, non‐randomized controlled study comparing proton‐beam therapy with hepatectomy for resectable HCC. Since proton‐beam therapy is a type of particle therapy, the results of this study are likely to provide valuable insights on the efficacy of CIRT for resectable HCC.

In conclusion, in our study, CIRT for primary, solitary, potentially resectable HCC was safe and associated with favorable clinical outcomes and acceptable liver damage according to the elevated ALBI score after treatment. CIRT might yield favorable clinical outcomes comparable to LR for patients with potentially resectable HCC; however, further large‐scale, prospective studies are needed to confirm our results.

## Disclosure

The authors have nothing to report.

## Ethics Statement

This study conformed to the ethical guidelines of the Declaration of Helsinki and was approved by the Institutional Review Board of Kanagawa Cancer Center (approval no. 2023 study 19).

## Consent

Informed consent was obtained from all patients to use their clinical data for this study.

## Conflicts of Interest

The authors declare no conflicts of interest.

## Data Availability

The datasets generated and analyzed during this study are available from the corresponding author upon reasonable request.
